# Systematic evaluation of a predator stress model of depression in mice using a hierarchical 3D-motion learning framework

**DOI:** 10.1038/s41398-023-02481-8

**Published:** 2023-05-25

**Authors:** Yu-Ting Tseng, Binghao Zhao, Hui Ding, Lisha Liang, Bernhard Schaefke, Liping Wang

**Affiliations:** 1 CAS Key Laboratory of Brain Connectome and Manipulation, Shenzhen-Hong Kong Institute of Brain Science, Shenzhen Institute of Advanced Technology, Chinese Academy of Sciences, Shenzhen, 518055 China; 2 Guangdong Provincial Key Laboratory of Brain Connectome and Behavior, the Brain Cognition and Brain Disease Institute, Shenzhen Institute of Advanced Technology, Chinese Academy of Sciences, Shenzhen, 518055 China

**Keywords:** Neuroscience, Diseases

## Abstract

Investigation of the neurobiology of depression in humans depends on animal models that attempt to mimic specific features of the human disorder. However, frequently-used paradigms based on social stress cannot be easily applied to female mice which has led to a large sex bias in preclinical studies of depression. Furthermore, most studies focus on one or only a few behavioral assessments, with time and practical considerations prohibiting a comprehensive evaluation. In this study, we demonstrate that predator stress effectively induced depression-like behaviors in both male and female mice. By comparing predator stress and social defeat models, we observed that the former elicited a higher level of behavioral despair and the latter elicited more robust social avoidance. Furthermore, the use of machine learning (ML)-based spontaneous behavioral classification can distinguish mice subjected to one type of stress from another, and from non-stressed mice. We show that related patterns of spontaneous behaviors correspond to depression status as measured by canonical depression-like behaviors, which illustrates that depression-like symptoms can be predicted by ML-classified behavior patterns. Overall, our study confirms that the predator stress induced phenotype in mice is a good reflection of several important aspects of depression in humans and illustrates that ML-supported analysis can simultaneously evaluate multiple behavioral alterations in different animal models of depression, providing a more unbiased and holistic approach for the study of neuropsychiatric disorders.

## Introduction

Central to most contemporary theories of depression is the notion that chronic stress is a major predisposing factor [1–3]. Social conflicts are considered a major source of stress for humans that contribute to the pathogenesis of depression [4, 5]. However, in most mammalian species, a number of different laboratory-based social stress paradigms involve hierarchies that are clearly defined with more pronounced and dominant in males than in females [6]. For example, social stress in rodents is mimicked using a chronic social defeat model (CSDS), which is based on the resident-intruder paradigm where the intruder eventually becomes subordinate to the unfamiliar territorial resident conspecific that defends its home cage [7]. A major drawback of this model is the difficulty of initiating a natural attack by the male territorial resident on female intruder mice. It is also for this reason, the vast majority of studies on depression involving social stress have been carried out using only male laboratory mice [7, 8]. Therefore, a better animal model of stress is needed so that mechanistic details of depression in females can be studied to the same degree as in males.

Predation is a major force behind natural selection that endowed animals with various innate and automatically activated defense behaviors and helped shape the evolution of nervous systems [9]. Although modern humans rarely become prey to other species, our defense system against predators may have been co-opted during the course of evolutionary history to also cope with social conflicts [10, 11]. The response to psychosocial threats in humans shares underlying neurocircuitry and physiology with the response to predators in rodents and primates [12–17]. The pathology of depression may involve common defense systems that evolved to deal with predators and social threats. Indeed, predator stress in rodents has been used as a potent stress stimulus to induce physiological and behavioral changes in male mice similar to those elicited by social threats, such as increased anxiety level [9]. However, it remains unclear whether chronic exposure to predator stress has the potential to induce depression-like behaviors in both male and female mice and replace sex-biased paradigms for future mechanistic studies and drug screening.

Canonical screening tests for evaluating depression-like behaviors in rodents, such as the tail suspension test and sucrose preference test are used to reflect the behavioral despair and anhedonia observed in human depression. However, the major drawbacks are that these tests provide a reductionist perspective of a complex disorder and that these behavioral changes lie on a continuum that cannot be easily sectionalized [18, 19]. On the other hand, automated behavioral analysis using ML has been applied to extract more complex behavioral patterns that can reflect an animal’s internal state. These behavioral patterns become altered after experimental manipulation or in disease models [20, 21]. Therefore, we aim to better annotate the overall ‘psychological state’ of the rodent using the Behavior Atlas (BeA) [22], a high-dimensional behavior mapping tool based on ML and automated 3D video capture.

In this study, we demonstrate that predator stress can induce depression-like behaviors in both male and female mice. Both predator stress and social stress models induced anhedonia in male mice, while the former elicited a higher level of behavioral despair and the latter elicited more robust social avoidance. Our results suggest that the CRS and CSDS models, while both sharing core characteristics of the human disorder, also have the potential to be applied during investigation of the divergent mechanisms underlying different depression subtypes. Furthermore, we used ML-assisted 3D video analysis of spontaneous behavioral patterns and compared these between the two types of stress. We also reveal the related patterns of spontaneous behaviors corresponding to the status measured by canonical depression-like behaviors. In addition, CRS-induced alterations in ML-observed spontaneous behaviors can be attenuated by the antidepressant fluoxetine. Our study illustrates that ML-supported analysis of behavioral fingerprints may enable us to gain a better understanding of an individual’s underlying mental state and can be particularly useful in the diagnosis and assessment of mental disorders.

## Materials and methods

### Animals

All experimental procedures were approved by the Animal Care and Use Committees at the Shenzhen Institute of Advanced Technology, Chinese Academy of Sciences. 8-week-old male and female C57BL6/J mice, 8–10-week-old male Sprague-Dawley rats (Beijing Vital River), and 4–6-month-old CD-1 male retired breeder mice (SiPeiFu Beijing) were used, all under a 12-h light/dark cycle with ad libitum access to food and water.

### Behavioral assays

Chronic predator stress (CRS) [11] and chronic social defeat stress (CSDS) [23] were performed to induce depression-like behavior in mice. The social interaction test (SI), sucrose preference test (SPT) and tail suspension test (TST) were used to asses depression-like behaviors, while the looming test was employed to evaluate innate defensive behaviors. The SI ratio was calculated as (time spent in the SI zone with a social target) / (time spent in the SI zone without a social target) [24]. Sucrose preference (%) was calculated as [sucrose solution consumed] / [sucrose + water solutions consumed] × 100 [24]. The time spent immobile was recorded by an observer blinded to experimental conditions in last 5 min of 6 min recording [25]. More details are provided in the supplementary information.

### 3D motion-capture system and behavior decomposition framework

The setup was similar to our previous study [22]. Briefly, cameras on the four sides of the apparatus synchronously record spontaneous behaviors of mice, then an ML-based method was used to automatically identify the behavioral phenotypes of mice. Unsupervised behavioral movement clusters were further recognized by supervised classification. Behavior fractions were calculated as the total time performing one type of behavior movement divided by the total time of all behavior movements, and a behavioral transition represents one type of behavior movement translated to another. More details are provided in the supplementary information.

### Statistical analysis

Sample sizes were determined according to previous studies [22, 24]. All relevant data were included in the analysis and mice were tested once, and no individual replicates were excluded. Before hypothesis testing, normality (Shapiro–Wilk normality test) and homoscedasticity (F-test) were verified. Statistics were performed in Prism 8.0 (GraphPad Software). Data are expressed as means ± SEM. Post hoc significance values were set as *p < 0.05, **p < 0.01, ***p < 0.001 and ****p < 0.0001. See also Table S1 for details.

## Results

### Chronic predator stress induces depression-like behaviors

**Fig. 1:**
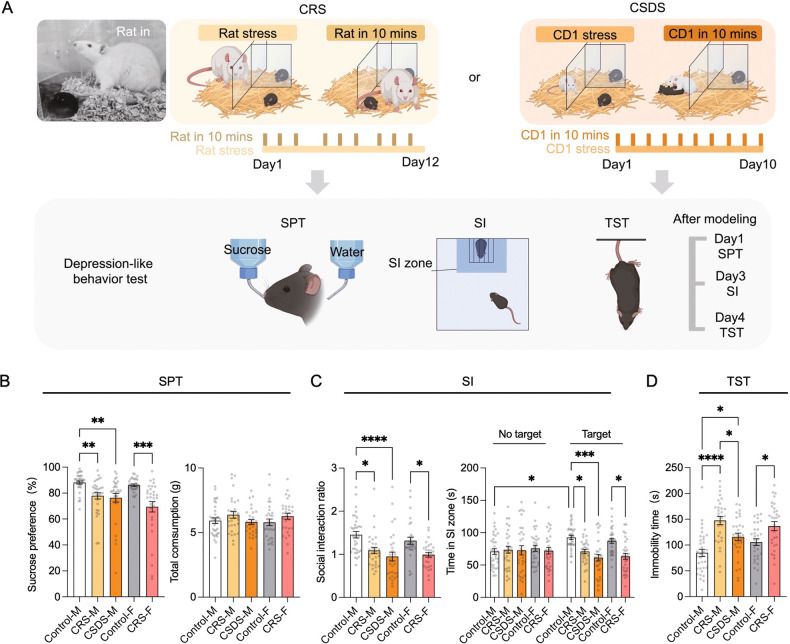
Depression-like behavior induced by chronic predator stress and social stress. **A** Schematic of the CRS and CSDS behavioral paradigms. Mice created with BioRender.com**. B**–**D** Sucrose consumption (%) and total consumption (**B**), SI ratio and durations in the SI zone without/with target mouse present (**C**), and immobility time (**D**). See Table S1 for statistical details.

Our previous work showed that mice exposed to chronic predator stress induced by a rat (CRS) displayed sleep pattern changes similar to those seen in human depression [11]. We subjected male and female mice to 12 consecutive days of predator stress and subsequently evaluated depression-like behaviors that are considered to reflect the core symptoms of human depression. Futhermore, CSDS generates a variety of behavioral changes (anhedonia, social withdrawal, despair) which are thought to reflect many of the core symptoms observed in depressed humans [5, 26–28]. This CSDS paradigm has been used extensively in basic and preclinical research to study social defeat stress and depression. Therefore, we also subjected another group of male mice to CSDS and compared depression-like behaviors between the CRS and CSDS groups (Fig. 1A) [23, 29]. Following CRS or CSDS in C57BL/6 J male mice, we performed a sucrose preference test to evaluate interest in rewarding stimuli [30]. We found that the male CRS group (CRS-M) and male CSDS group (CSDS-M) exhibited a significantly lower sucrose preference than a male stress-naïve control group (Control-M in figures), suggesting that both CRS and CSDS can promote anhedonia-related behavior (Fig. 1B). Furthermore, female mice subjected to CRS (CRS-F) also exhibited a lower sucrose preference than female stress-naïve controls (Control-F) (Fig. 1B). All groups had similar total fluid intake (Fig. 1B). Next, we used a social interaction (SI) test to compare CRS- and CSDS-induced deficits in sociable behavior [31]. Compared to Control-M mice, CRS-M and CSDS-M mice had lower SI ratios (Fig. 1C). This result indicates that both CRS and CSDS lead to social avoidance in C57BL/6 J male mice. CRS induces similar social withdrawal behaviors in C57BL/6 J female mice (Fig. 1C). It is noteworthy that in C57BL/6 J male mice, CSDS produces stronger social avoidance than CRS. Lastly, behavioral despair was assayed using the tail suspension test (TST), in which animals were exposed to an inescapable aversive situation. In this situation, mice originally attempt vigorous escape but switch to a passive coping state after prolonged behavioral challenge [32]. Interestingly, we found that mice subjected to CRS displayed a significantly higher level of passive coping compared to both stress-naïve and CSDS groups (Fig. 1D). Together, these data demonstrate that both CRS and CSDS drive depression-like behaviors in male mice, and CRS also induces such behavior changes in female mice.

### Anhedonia phenotypes induced by chronic predator stress correlate with an increased defensive state

High perceived stress level, an individual’s appraisal of their inability to cope with recent threats from stressors, is shown to be a critical etiological factor in the pathogenesis of depression [33, 34]. Given this, we investigated the premise that defensive behavior in response to perceived stress may be associated with depression-like behaviors. We used the looming assay (rapidly expanding dark overhead spots mimicking an approaching predator) to evaluate levels of defensive behavior [35, 36]. Both male and female mice subjected to CRS displayed enhanced defensive behavior (Fig.S1A-S1B). Interestingly, correlation analysis showed sucrose preference was negatively correlated with the time spent hiding in the refuge after mice experienced CRS (Fig.S1C-S1H).

### Systemic evaluation of behavior profiles following CRS and CSDS using a 3D motion-capture system

**Fig. 2:**
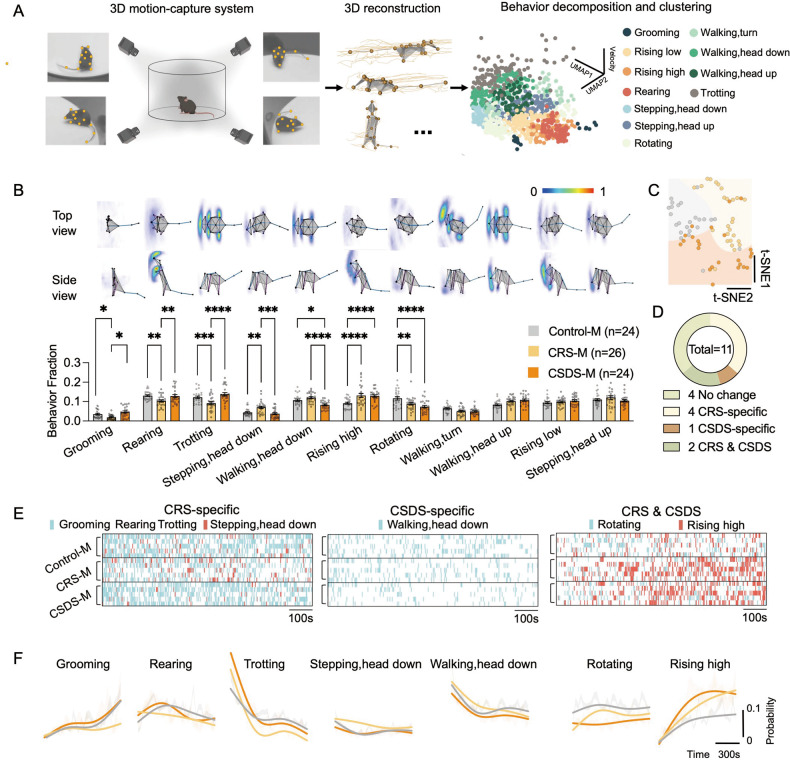
Chronic predator and social stress impact spontaneous behavior profiles. **A** Left: Pipeline of mouse behavior recording and analysis via 3D-motion learning framework. Right: 3D scatter plot illustrates the spatiotemporal feature space of the components of 11 behaviors of 1 mouse. **B** Up: averaged skeletons of 11 behavior movements. Down: comparison of the fractions of different movement types found in Control-M, CRS-M and CSDS-M mice. **C** Two-dimensional embedding of behavior fractions by t-SNE. The SVM decision boundary was identified by gaussian Kernel function. **D** Comparison of altered behavior movement numbers between CRS-M and CSDS groups. **E** Raster of the behavior movements. **F** Probability of behavior movements across time. See Table S1 for statistical details.

To obtain detailed annotation of naturalistic behaviors in mice following CRS and CDS, we took advantage of a hierarchical 3D-motion learning framework and obtained behavioral phenotypes from both groups [22, 37]. Since CSDS was mainly used to drives depression-like behaviors in male mice, we first measured male mice so that behaviors induced by CRS and CSDS could be systematically compared. Male mice were divided into three groups: no treatment, CRS, or CSDS (Control-M, CRS-M, and CSDS-M, respectively). Spontaneous behaviors of these groups were recorded for 15 min (Fig. 2A). The data were automatically analyzed using the ML-based behavior analysis framework (BeA) [22], which extracted 40 behavioral motifs with unsupervised clustering (Fig. S3). We obtained 11 major behavior types from 74 animals by manually grouping these 40 behavioral motifs (Fig. 2A and Table 1). These behaviors included *grooming*, two types of rising (*rising high* and *rising low*), *rearing*, two types of stepping (*stepping, head down* and *stepping, head up*), *rotating*, three types of walking (*walking, turn*, *walking, head down*, and *walking, head up*), and *trotting*. We found several aligned and non-aligned differences in the 2 stress groups (CRS-M and CSDS-M) compared to controls in all 11 behavioral modules (Fig. 2B). In the CRS-M group, there were significant fewer instances in *grooming*, *rearing*, *trotting*, but more *stepping, head down*, and these are thereby defined as CRS-specific behaviors. Only the *walking, head down* behavior was significantly lower in the CSDS-M group and is thus defined as CSDS-specific. In addition, two behavioral features, *rising high* and *rotating*, showed similar changes in the CRS-M and CSDS-M groups and are thereby defined as both CRS and CSDS (Fig. 2D–F).

**Table 1:** Definition of the behavior movements for manual labeling.

Behavior	Definition
Grooming	Mouse cleans body and fur by licking and scratching.
Rising low	Mouse rises from the ground to stand on its hind legs with bent back, showing less body lift and lower head height relative to the ground compared to the ‘rise high’ behavior.
Rising high	Mouse rises from the ground to stand on its hind legs with bent back, showing more body lift and higher head height compared to the ‘rise low’ behavior.
Rearing	Mouse stands on hind legs with straight back.
Stepping, head down	Mouse moves a short distance at a slow speed, with its head towards the ground.
Stepping, head up	Mouse moves a short distance at a slow speed, with its head facing forward.
Rotating	Mouse turns around with slow speed and moves a short distance.
Walking, turn	Mouse moves a far distance at a moderate speed, along with body turns.
Walking, head down	Mouse moves a far distance at a moderate speed, with its head towards the ground and shows fewer movements.
Walking, head up	Mouse moves a far distance at a moderate speed, with its head facing forward and shows more movements.
Trotting	Mouse moves a far distance at a fast speed.

Next, we examined whether these behavioral features can effectively discriminate between the three groups. To do this, we embedded the behavioral fractions of 11 behavior movements from each mouse into a 2D t-SNE manifold. We found that individuals of the Control-M, CRS-M, and CSDS-M could be classified into three clusters by a Support Vector Machine (SVM) classifier (Fig. 2C). An organism’s behaviors can be decomposed into discrete, reproducible motifs and they can be described in terms of a small set of stereotyped motifs [38–40]. Therefore, we further quantified the transitional dynamics of these 11 behavioral features, whilst aiming to retain each animal’s full behavioral complexity. We found that the CRS-M group had significantly fewer behavioral transitions compared to the Control-M and CSDS-M groups, implying an impairment in behavioral flexibility (Fig. S3A and S3E). Both CRS-M and CSDS-M groups displayed various transitions between bouts of behavioral features which differed from the Control-M group, sometimes in a divergent manner (Fig. S3B, S3D and Fig. S4). Furthermore, we found that embedding the total 106 behavioral transitions into a 2D t-SNE manifold allowed for a more effective discrimination between the Control-M, CRS-M, and CSDS-M groups (Fig. S3C). These experiments demonstrate that, although sharing common features, each stress type elicits a specific pattern of behavior in mice.

### Spontaneous behavior profiles correlate with depression-like behaviors

**Fig. 3:**
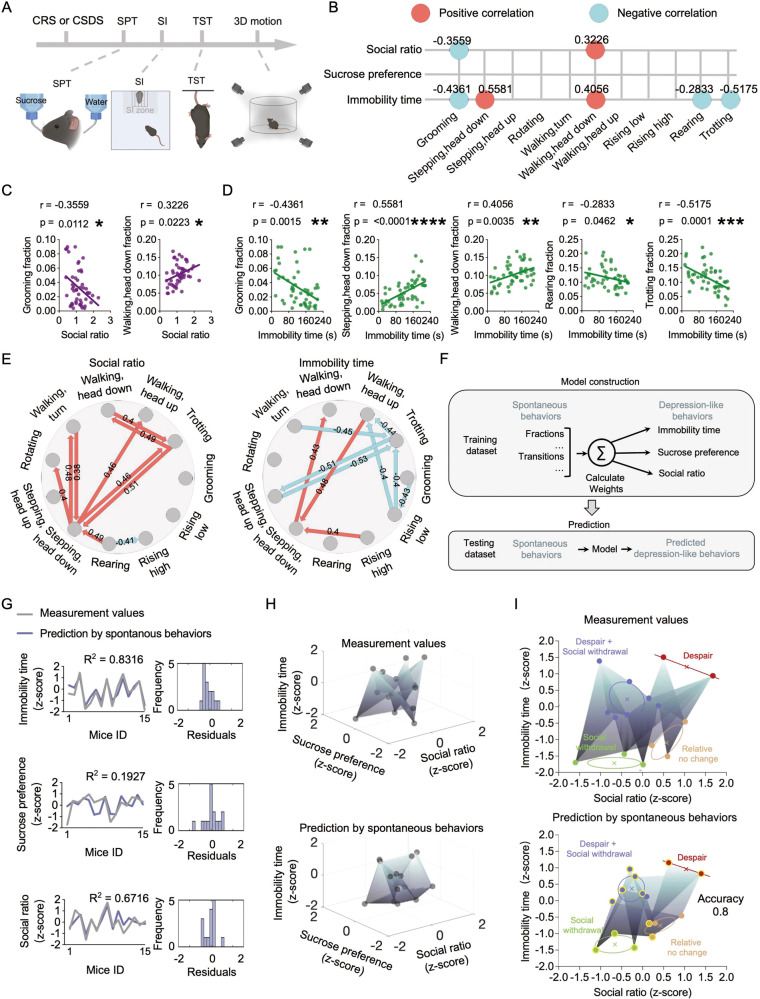
Stress-induced depression-like behaviors are correlated with spontaneous behavioral profiles. **A** Schematic diagram of the behavioral tests. **B** Summary of the correlation analysis between depression-like behaviors and the fraction of each spontaneous behavior. The numbers above the dots indicate the correlation coefficient. **C**, **D** Representative correlation analysis between depression-like behaviors and the fraction of each spontaneous behavior. **E** Summary of the correlation analysis between behavioral transitions and two depression-like behaviors. The arrows between two behavioral states indicate the direction of transition. The numbers above the arrow indicate the correlation coefficient. See also Fig. S6. **F** Pipeline for ElasticNet linear regression model construction and prediction. **G** Model prediction of depression-like behaviors (left) and residual histograms (right). Coefficient of determination (R²) were labeled. **H** Measurement (up) and Prediction (down) values of depression-like behaviors were shown in the ‘Immobility time × sucrose preference × social ratio’ space. **I** K-means clustering (*k* = 4) of measurement (up) and prediction (down) values of immobility time and social ratio. Samples belonging to the same cluster in measurement and prediction clusters are labeled with yellow circles in the prediction data. Pearson correlation analysis.

Although chronic stress caused both depression-like behaviors and changes in spontaneous behavior features, it was still unclear whether there is a correlation between them. Therefore, we evaluated the correlation between depression-like behaviors and behavior profiles using Pearson correlation analysis. Following either CRS or CSDS, mouse behavioral features were measured using the 3D motion-capture system and depression-like behaviors were also tested using the SI, SPT, and TST paradigms (Fig. 3A). Interestingly, we found that the fraction of *grooming* in mice positively correlated with social avoidance in the SI test and negatively correlated to behavioral despair in the TST test (Fig. 3B–D and Fig. S5). Furthermore, *stepping, head down* and *walking, head down* were positively correlated with behavioral despair and *walking, head down* was negatively correlated to social avoidance. *Rearing* and *trotting* were negatively correlated with behavioral despair (Fig. 3C, D). To further explore the correlation between behavioral transitions and depression-like behaviors, we matched the 106 types of transitions with depression-like behaviors (Fig. 3E and Fig. S6). We found that SI ratio and immobility time were correlated with 10 behavioral transition types, whereas no transitions correlated with sucrose preference. Given that spontaneous behavior fractions and transitions were correlated with depression-like behaviors, we examined whether we could use spontaneous behavior to predict depression-like behaviors. Fifty samples were partitioned into separate training and testing datasets. A linear model was trained using spontaneous behavior fractions and transitions that were significantly correlated with depression-like behavior to fit immobility time, sucrose preference and social ratio. After training, the model was applied to predict depression-like behaviors using spontaneous behavior fractions and transitions as predictors in the testing dataset (Fig. 3F). We found that prediction values fit with immobility time and social ratio, but poorly fit with sucrose preference (Fig. 3G). Additionally, the three-dimensional space comprised of depression-like behaviors revealed that, relative to the sucrose preference, the prediction and measurement data exhibited a more comparable distribution of immobility time and social ratio dimensions data points (Fig. 3H). To further evaluate this model, we performed K-means clustering to identify featured clusters (Fig. 3I). Based on the variations in the performance of behavior despair and social withdrawal across these clusters, we identified the clusters as ‘Relatively no change’, ‘Social withdrawal’, ‘Despair’ and ‘Despair + Social withdrawal’. The classification accuracy of the predicted data in these four groups was found to be 80 %, indicating that spontaneous behaviors can predict depression-like behaviors. Together, spontaneous behavioral profiles as well as the defensive level of an animal has the potential to identify depression-like symptoms.

### Female mice display spontaneous behavioral changes following CRS similar to male mice

**Fig. 4:**
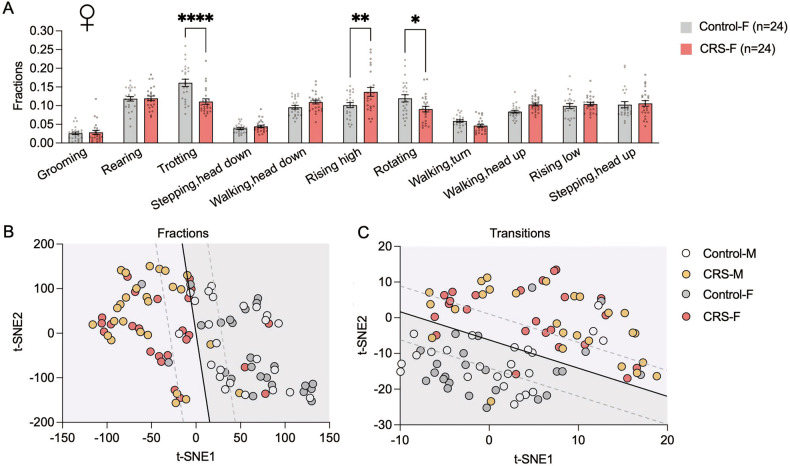
Spontaneous behavioral changes elicited by predator stress in female mice. **A** Comparison of the fraction of spontaneous behavioral types of Control-F and CRS-F mice. **B**, **C** Two-dimensional embedding of behavioral fractions (**B**) and transitions (**C**) by t-SNE, Solid lines are the decision boundary identified by SVM. See Table S1 for statistical details.

Although males and females do not differ in overall depression-like behaviors following CRS, it is not clear whether they differ in the finer behaviors tracked by the 3D motion‐capture system. Therefore, we compared spontaneous behaviors in the Control-F and CRS-F groups to examine the impact of CRS on female mice. Female mice following CRS showed similar significant differences in *trotting*, *rising high* and *rotating* fractions as male mice following CRS compared to their respective control groups (Fig. 4A). To compare behavioral patterns of female and male mice, we embedded the behavioral fractions from each sex into a 2D t-SNE manifold, and found that male and female mice before CRS showed similar distributions and that the distribution patterns of both sexes tended to move in the same direction in t-SNE feature space following CRS. Furthermore, a linear classifier effectively classified pre-CRS mice with post-CRS mice but was not able to distinguish sex, indicating that when it comes to overall behavioral changes, both male and female mice exhibit similar trends (Fig. 4B). In addition, CRS-F mice displayed significantly fewer behavioral transitions compared to Control-F, revealing a common feature where both female and male mice have an impairment in behavioral flexibility after CRS (Fig. S7C). The majority of the behavioral transition types showed similar trends in males and females (Fig. S7A–S7D). In addition, we embedded all behavioral transitions into a 2D t-SNE manifold and found that the linear classifier could effectively classify whether a mouse had experienced CRS, although it could not differentiate between male and female mice (Fig. 4C). Together, these data suggest that CRS-induced behavioral changes using an automated 3D-motion capture system are similar between sexes.

### Fluoxetine treatment eliminates spontaneous behavior changes following CRS

**Fig. 5:**
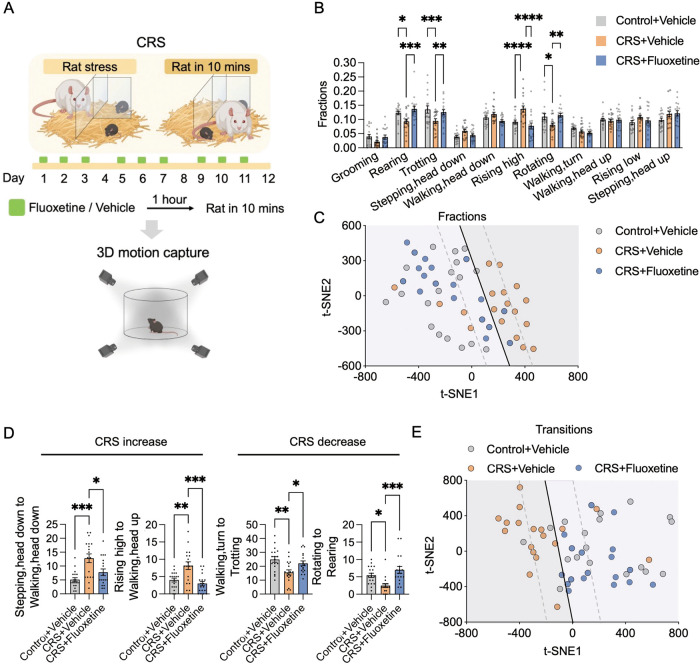
Fluoxetine treatment eliminates behavioral profiles altered by chronic predator stress. **A** Schematic diagram of the behavioral tests. **B** Comparison of the fractions of each spontaneous behavioral type in Control+Vehicle, CRS+Vehicle and CRS+Fluoxetine mice. **C** Two-dimensional embedding of all behavioral fractions by t-SNE. **D** Representative behavioral transitions observed in the Control+vehicle, CRS+vehicle and CRS+Fluoxetine groups. **E** Two-dimensional embedding of all behavior transitions by t-SNE. See Table S1 for statistical details.

Behavioral measures intended for use as a new animal model of depression should initially satisfy the criteria of having strong predictive validity [41, 42]. Therefore, we further examined whether the behavioral changes induced by CRS can be attenuated by fluoxetine, a selective serotonin reuptake inhibitor (SSRI) for depression treatment [43, 44]. Fluoxetine treatment (20 mg/kg/day in 0.2 ml saline) was applied every day during the period when mice were subjected to CRS (Fig. 5A). The effects of fluoxetine treatment on the spontaneous behavior induced by CRS were inferred by the differences in behavioral fractions and transitions between treatment and control groups (Fig. 5B–E). In both male and female mice, chronic fluoxetine treatment (CRS+Fluoxetine) significantly eliminated the behavioral changes following CRS (CRS+Vehicle) (Fig. 5B and D). In addition, we embedded the fractions of 11 behaviors and 106 types of transitions of each mouse into a 2D space by t-SNE (Fig. 5C and E). We found that mice with or without fluoxetine treatment could be classified using a linear classifier. There was a similar distribution of data points between the CRS+Fluoxetine and stress-naïve control groups (Control+Vehicle). Both Control+Vehicle and CRS+Fluoxetine groups exhibited distinctly different distributions with CRS+Vehicle. In summary, the predictive validity criteria suggest that the spontaneous behaviors in CRS mice may be used as a reliable measure of depression.

## Discussion

Modeling human psychopathologies by means of laboratory animals requires several criteria, and among these, construct, face, and predictive validity have been given priority [45–47]. CSDS is considered a valid animal model of depression as it fulfills these validity criteria [48, 49]. For example, most common stressors in humans are psychosocial in nature and the neural correlates of processing social stress have been verified in rodents, establishing construct validity [50]. CSDS is also a model with high face validity, because it produces specific behaviors resembling the signs and symptoms of humans with depression, such as anhedonia, social avoidance, and despair [51–53]. However, not only is the CDS paradigm challenging to model in female laboratory mice, but it also has the potential for skin wounds on the test mouse as a result of attacks, which can impair health and preclude continued inclusion in the experiment [5]. The major advantages of our CRS model are that it does not involve physical stress, and that predator stress can be conducted on both sexes. The CRS model does not only have good face validity, but its predictive validity is also demonstrated by a lack of behavioral changes after chronic treatment with fluoxetine. The core components of defensive responses to predator stress at the circuit level are highly conserved across mammalian species [54], suggesting good construct validity of the CRS model. Interestingly, despite the commonalities between CRS and CSDS, there are some notable differences. There tend to be higher levels of behavioral despair following CRS, while CSDS elicits higher degrees of social avoidance. This suggests that these two models can be used for different representations of depression symptomatology. Clinical studies have identified two subtypes of major depressive disorder, atypical depression and melancholic depression [55–57]. Atypical depression is characterized by symptoms like interpersonal rejection sensitivity, which may lead to social withdrawal and avoidance, whereas melancholic depression typically includes symptoms like psychomotor retardation which is shown as loss of responsiveness to the individual’s environment. Patients with different subtypes of depression may exhibit differences in endocrinology, neurocircuitry changes, as well as differences in response to treatment, which requires suitable animal models to explore the mechanisms underlying different depression subtypes [58]. Gratifyingly, the CRS model closely resembles symptoms of melancholic depression, whereas the CSDS model captures some of the symptoms more frequently found in atypical depression, suggesting that these models have potential to be applied in studying the mechanisms of different depression subtypes. In summary, our study illustrates that the CRS-induced phenotype in mice is a good reflection of behavioral aspects of depression in humans, as currently understood.

This is the first study to dissect the correlation of animal defensive behavior with the depressive state. We found that time spent hiding in the nest in response to predator stimuli was correlated with the level of anhedonia in the sucrose preference test. This discovery indicates that hiding behavior, but not escape itself, may reflect the depression-like state in mice and suggests an adaptive function for this state. Given that animals being preyed upon often decrease feeding activity to take cover and avoid detection by predators [59], prolonged hiding and reduced reward gained from palatable food may constitute a coordinated response to predator stress, effectively reducing the time and energy allocated to foraging in a dangerous environment. Whether this lowered reward responsivity may have become maladaptive in humans who suffer from sustained stress or whether it constitutes an adaptive change in motivation [60] remains unkown. In either case, our results clearly suggest that anhedonia and increased defensive level expressed as hiding in a shelter following CRS may emerge from shared neural mechanisms. Furthermore, we also found that the group of mice subjected to CRS showed decreased struggling in the tail suspension test, but enhanced escape in the looming test. These seemingly contradictory results support the hypothesis that prior experience of CRS leads to optimized decision-making processes rather than a state of generalized “learned helplessness” [61]. Mice may learn to decide when to escape and when to give up, depending on a more realistic perception of control during stressful events. This is consistent with the still controversial view that, in humans, depressed individuals frequently show more accurate judgment of their ability to influence an outcome than healthy individuals [62], termed “depressive realism”.

Animals have complex behavioral patterns, the contents of which manifest brain function as the result of the manifold interactions of gene regulatory networks and neural circuits. Overly simplistic behavioral assays with low dimensionality can lead to significant difficulty to link drug effects to molecular mechanisms in complex diseases such as depression [18, 21]. ML tools have enabled the extraction and classification of more complex behavioral patterns, and the identification of pattern perturbation by drugs and disease models [20]. Furthermore, bodily expression and emotional processing are closely connected [63], and clinical studies have revealed that depressed individuals exhibit a change in body movements and posture [57, 64, 65]. For example, depressed patients often display a decrease in spontaneous movements and body postures[66], and measuring these behaviors can supply a wealth of information in clinical diagnosis [67–69]. In this study, we showed that mice following predator or social stress displayed increased rising and decreased rotating behaviors. Rising is usually associated with exploration and foraging, but may also be a sign of anxiety or fear, as rodents may stand upright in response to a perceived threat [70]. Rotating is a spontaneous cycling action that requires animals to be able to flexibly control their bodies. Interestingly, studies have shown that patients who are depressed often showed rigid postures such as reduced arm swing and head movements [64, 65], indicating that decreased rotating behavior may also be an expression of behavior rigidity in depression that manifests as a reduced range of motion. It may be the case that a mechanism common to both CRS and CSDS modulate these two behavioral changes, rising and rotating. Furthremore, we successfully captured the spontaneous behavioral differences between CRS and CSDS models, illustrating that ML-supported analysis can simultaneously evaluate multiple behavioral alterations in different animal models of depression.

With regards to testing for depression-like behaviors in rodents, canonical screening tests (such as the TST, SI and SPT) are used mainly to reflect specific clinical symptoms [62]. Our results indicate that CRS induces more behavior despair whereas CSDS triggers more social avoidance. Correlation analysis between ML-observed spontaneous behaviors and depression behavior indicators revealed that head-down movements showed a positive correlation with behavioral despair, whereas rearing and trotting showed a negative correlation. Interestingly, depressed patients show increased head-down postures and reduced behavior movements and speeds [64, 65], which is consistent with our observations. These results all suggest that changes in the motor network coincide with the development of behavioral despair. Moreover, we found that grooming was most strongly correlated with social avoidance. In line with our findings, it has been observed that mice lacking ephrin receptors display both increased self-grooming and impaired social interaction [71]. Furthermore, a shared mechanism has been shown to control these two behavioral phenomena, as stimulation of neurons in the posterior dorsal part of the medial amygdala can induce repetitive self-grooming in mice and suppress social interaction [72]. Furthermore, we showed that depression-like behaviors can be predicted by patterns of spontaneous behaviors. This evidence suggests that studying spontaneous behavior will help to explain the implications of behaviors in more constrained tasks. Together, our ML-based approach provides predictive classifiers for social and despair behavior in rodents, and overcomes several limitations in the classical behavioral paradigms including dependence on human observers and a heavy susceptibility to confounders [73].

We acknowledge several research limitations in our study. First, our ML-observed approach is performed in single mice and outside their normal living environment [74]. As Shemesh et al. proposed, it would be interesting to assess potential changes in complex behavioral patterns following stress in an enriched environment that include essential resources, potential mating partners, and social competitors [74]. This would require an automatic setup that enables the tracking of group-housed mice individually in complex 3D environments [74, 75]. Second, CRS-induced changes in behavioral fractions using ML-observed behavior were similar between sexes despite the fact that depression affects females around twice as often as it does males [76]. In response to environmental stimuli, such as predator odor, mice can display fear-related behaviors that are not composed of new syllables but rather of new sequences of the same syllables [77]. Therefore, future study should be directed towards investigations comparing the behavioral sequences between sexes and longitudinally characterizing the behavioral patterns of both female and male mice several weeks after the end of the stress paradigm.

## Supplementary information

Supplemental Information

## Data Availability

Behavior atlas (https://behavioratlas.tech//) was used for spontaneous behavior analysis, all other scripts will provide upon request.
