# Supplementary material for: Systematic evaluation of a predator stress model of depression in mice using a hierarchical 3D-motion learning framework
Source: Transl Psychiatry. 2023 May 25;13:178. doi: 10.1038/s41398-023-02481-8 (PMC10213065; doi:10.1038/s41398-023-02481-8)
Supplement: Supplementary file 1 — Supplemental Information [file 41398_2023_2481_MOESM1_ESM.pdf]

Systematic evaluation of a predator stress model of depression in mice using a hierarchical 3D-motion learning framework

Supplemental Information

Supplemental Methods

Fig. S1-S7

Table. S1

Supplemental Methods

Behavioral assays

Chronic predator stress (CRS)

The CRS paradigm was performed according to a previous study in our laboratory [1]. Briefly, a walled arena was divided into three chambers by transparent plexiglass separators (two mice zones and one rat zone). A male SD rat was placed in the rat zone for up to 12 days to provide sensory stress to mice throughout this period. The SD rat was transferred to the mice home chamber for 10 minutes, allowing physical interactions between them every day except day 4, 8 and 12. During this period of contact, the experimenter removed the mouse immediately when the SD rat showed any signs of aggression.

Chronic social defeat stress (CSDS)

The CSDS protocol used was modified from previously described protocols [2]. In brief, the same apparatus used to perform CRS was used for CSDS. Before the experiment, retired male breeder CD-1 mice were habituated to one chamber and screened for aggressive behavior. Over the following 10 consecutive days, C57BL6/J mice were transferred to the CD-1 chamber and were physically defeated by CD-1 aggressor mice for 10 minutes each day. After each physical interaction, mice were housed individually in the other two chambers.

Social interaction test (SI)

An open-field test box (50 cm × 50 cm × 50 cm) containing an empty metal cage (9 cm × 9 cm × 13 cm) on one side was used to measure social interaction. On the first 150 s

trial, an experimental mouse was allowed to explore freely in the open-field arena with the empty metal cage (defined as the “No Target” phase due to no social target mouse). Subsequently, an unfamiliar mouse of the same sex and age was placed in the metal cage, and the experimental mouse was allowed to explore for another 150 s (defined as the “Target” phase). The time spent interacting with the metal cage with or without a social target was measured using ANYMAZE video-tracking system (Stoelting, IL, USA). The social interaction ratio was calculated as (time spent in the SI zone with a social target) / (time spent in the SI zone without a social target).

Sucrose preference test (SPT)

Mice were habituated for two consecutive days to two 80-ml tubes (with fitted ball-point sipper) filled with water. After habitation, mice were exposed to one tube of 1% sucrose and one tube of water for a further 2 days. During this period, the weight of two tubes were recorded before the experiment began, and at the end of the first and second day. The position of the tubes was interchanged daily to avoid any side preference. Sucrose preference (%) was calculated as (sucrose solution consumed] / [sucrose + water solutions consumed] × 100.

Tail suspension test (TST)

Each mouse was suspended by fixing its tail with tape, leaving the mouse 50 cm above the surface. A plastic tube was placed over the tail of mouse to prevent tail climbing behavior. The time spent immobile (immobility time) was recorded by an observer blinded to experimental conditions; this was defined as the total time in which the body, including all limbs, did not move within last 5 minutes of the 6-minutes tail suspension.

Looming stimuli

The apparatus consisted of a 40 × 40 × 30 cm closed Plexiglas box and an adjacently connected rectangular alley as a refuge. An LCD monitor was placed on the ceiling to present looming stimuli, which consisted of an expanding disc mimicking the approach of predator. During looming tests, the mouse was allowed to freely explore for 5 minutes. Following this, looming stimuli were presented for 2 to 3 trials with inter-trial intervals of 3 minutes. Videos of mouse behavior were manually analyzed by an observer blinded to experimental conditions using Adobe Premiere. The following parameters were calculated: (i) latency to flight (s): time between the onset of looming stimulus and the start of escape behavior, (ii) return time (s): time between the onset of looming and returning to refuge, and (iii) time spent in the refuge (s): time between the mouse entering the refuge following looming stimulus and the mouse leaving the refuge. All animals are simultaneously randomized to the treatment groups without considering any other variable. The assessors performing manual scoring of behavioral experiments were blinded to the experimental conditions in all tests described above.

Unsupervised behavior embedding and linear classification

To visualize high-dimensional behavioral movement fractions and transitions, we embedded 11 behavioral movement fractions or 116 types of transition numbers into a 2D space using a t-distributed stochastic neighbor embedding (t-SNE) manifold. Based on this 2D space, the support Vector Machine (SVM) decision boundary was identified by Gaussian Kernel function to classify groups [3].

Linear Regression Model and K-means clustering

ElasticNet linear regression model was used to predict depression-like behaviors from spontaneous behavioral data. A total of 50 samples were randomly divided into a training set (35 samples) and a test set (15 samples). Measurement data of spontaneous behaviors and depression-like behaviors in the training set were used to train the model using the 'lasso' function in MATLAB. Data from the measurement of spontaneous behaviors in the test set were used to predict depression-like behaviors. Coefficient of determination (R^2) was used to determine whether the predicted depression-like behaviors fit the measurement values using a value between 0 and 1, with higher values indicating a better prediction performance. K-means clustering was used to cluster measurement and prediction values separately. Classification accuracy was computed as the total number of correctly classified predictions divided by the total number of measurement data.

3D motion-capture system and behavior decomposition framework

Each mouse was allowed to freely move in a transparent circular open field (50 cm diameter). Four cameras (Intel RealSense D435) were placed, one on each on the four sides of the apparatus to synchronously record 15 minutes of behavior. Synchronized videos were obtained using multi-view cameras in a 3D motion-capture system. Subsequently, a machine-learning-based method called Behavior Atlas (BeA) was used to automatically identify the behavioral phenotypes of mice. During the BeA process, mouse body parts recorded on each camera were tracked using Deeplabcut training to obtain separate 2D skeletal trajectories, and then 3D skeletons were reconstructed by

integrating skeletal trajectory video data acquired from the four cameras. When presenting the mouse skeletons, poses of body parts across time were calculated to generate averaged skeletons. The distribution and intensity of movements (MI) are indicated by heatmaps overlaid on the skeleton, MI are scaled from 0–1 and shown in arbitrary units (a.u.). Then, behavior decomposition and unsupervised clustering were performed based on the 3D skeleton data stream to obtain behavior movement clusters. The complex mouse behaviors included locomotion information, represented by velocity and non-locomotor movement (NM) using limbs or organs without movement of the torso, such as grooming, and dynamic time alignment kernel (DTAK), used to measure the similarity between non-locomotor movement segments. High-dimensional NM features were embedded into 2D NM space using uniform manifold approximation and projection (UMAP), which constituted the 3D behavior feature space together with the velocity parameter. Based on 3D behavioral features, unsupervised clustering was applied to cluster behavioral types. The behavioral movements were further recognized by supervised classification. Supplementary Figure 2 shows all 40 unsupervised behavioral movement clusters, which were further manually combined to obtain 11 behavior movements.

Supplemental Reference

1 Tseng YT, Zhao B, Chen S, Ye J, Liu J, Liang L, et al. The subthalamic corticotropin-releasing hormone neurons mediate adaptive REM-sleep responses to threat. Neuron. 2022;110(7):1223-39.e8.

2 Golden SA, Covington HE, 3rd, Berton O, Russo SJ. A standardized protocol for repeated social defeat stress in mice. Nat Protoc. 2011;6(8):1183-91.

3 Chang C-C, Lin C-J. LIBSVM: A library for support vector machines. ACM Trans Intell Syst Technol. 2011;2(3):Article 27.

Supplemental Figures

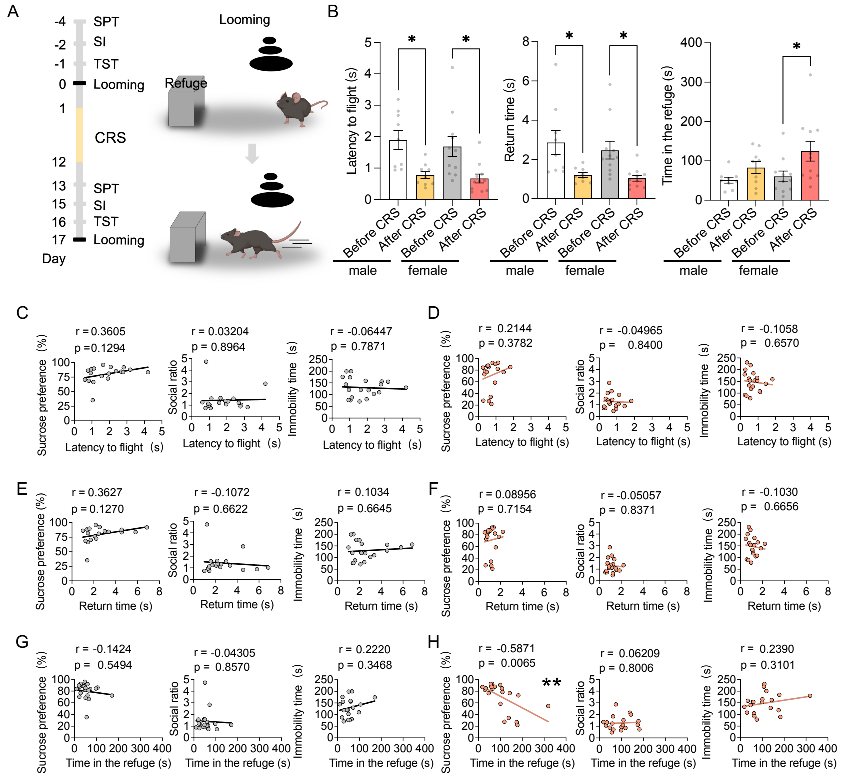

Supplementary Figure 1 Anhedonia phenotypes correlate with defensive responses

A Schematic diagram of the behavioral tests. Mice created with BioRender.com. **B** From left to right: latency to flight, return time to the refuge, and time spent hiding in the refuge following exposure to looming stimuli (n=9 for male mice, n=11 for female mice). Data represented as means ± SEM. Kruskal-Wallis test followed by Dunn's post hoc multiple comparisons test, $*p < 0.05$. **C, E, G** Pearson correlation analysis of defensive behaviors with sucrose preference, social ratio or immobility time before CRS. **D, F, H** Pearson correlation analysis of defensive behaviors with sucrose preference, social ratio or immobility time after CRS (r, correlation coefficient). $**p < 0.01$.

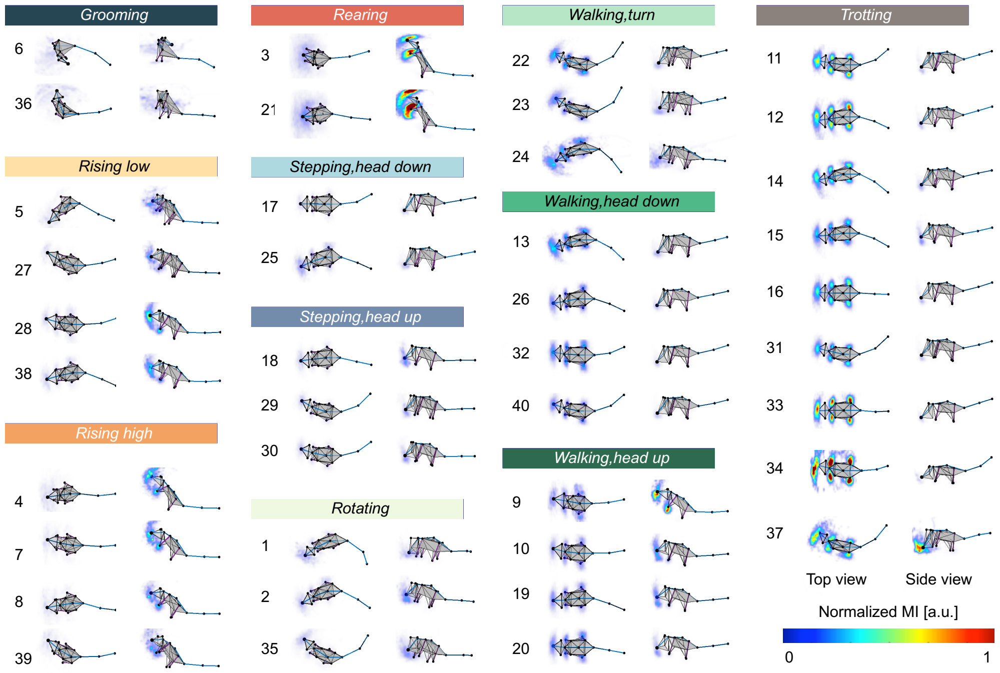

Supplementary Figure 2. Visualization of behavioral kinematics after unsupervised clustering.

Averaged skeletons and normalized moving intensity (MI) of 40 behavioral phenotypes obtained through unsupervised clustering were shown. The poses of body parts across time were calculated to generate averaged skeletons (Solid lines), the distribution and MI were indicated by heatmaps overlaid on the skeleton, the MI are scaled with 0–1 and shown in arbitrary unit (a.u.).

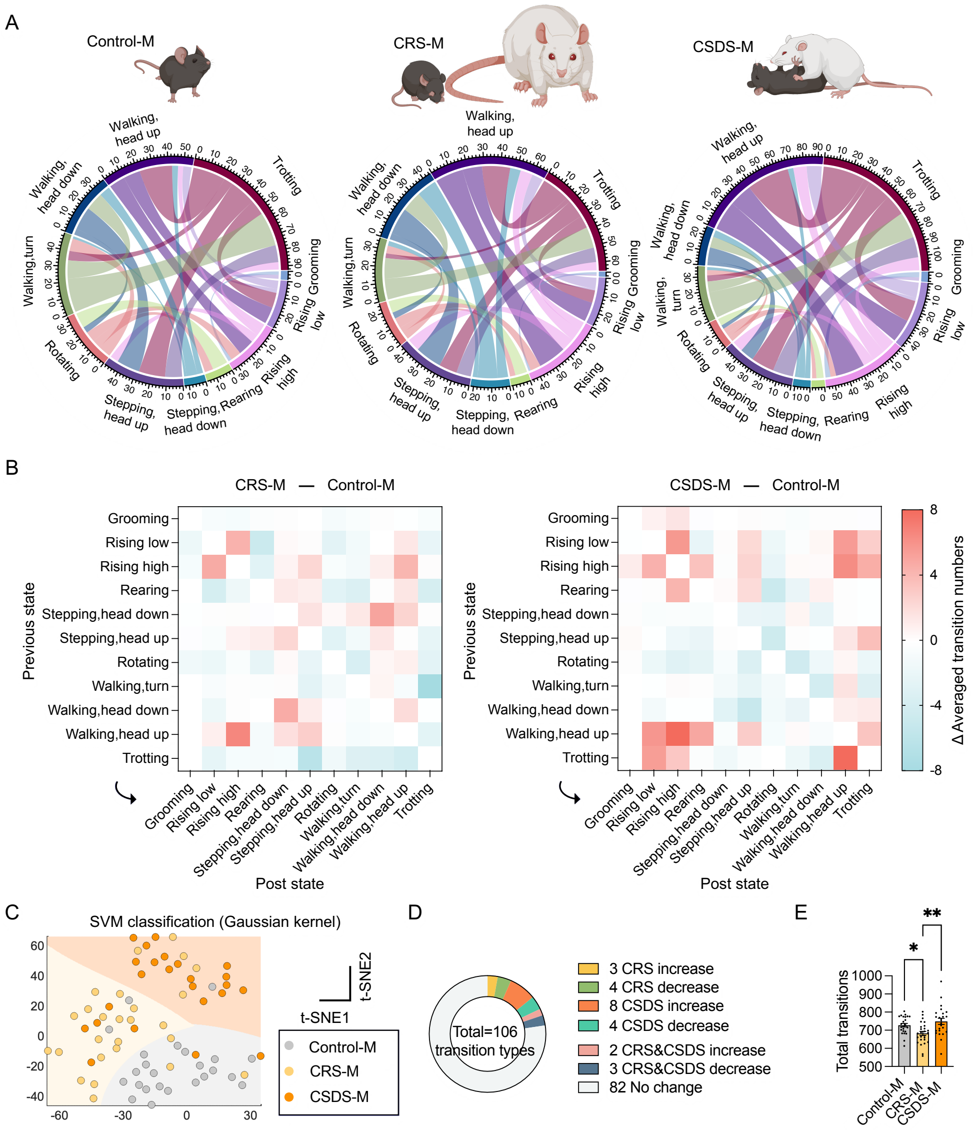

Supplementary Figure 3. Alteration of behavior transitions after chronic stress.

A Representative behavioral state transitions observed in the Control-M (left), CRS-M (Middle) and CSDS-M (right) groups. Each connecting line indicates the transition from the previous state to the post state and their colors are labeled according to the previous state. Mice created with BioRender.com. **B** Comparison of the numbers of behavioral transitions between CRS-M and Control-M groups (left), and between CSDS-M and Control-M groups (right). The previous behavioral states are listed on the Y-axes and the post states on the x-axes. Δ Averaged transition numbers were

calculated using the averaged numbers of each transition types in the CRS-M group subtracted from the Control-M group (left), or in the CSDS-M group subtracted from the Control-M group (right). Red and blue colors indicate increased and decreased transition numbers, respectively. A total of 106 transition types were observed. **C** Two-dimensional embedding of all transition numbers of Control-M (grey dots), CRS-M (light orange dots) and CSDS-M (dark orange dots) group mice by t-SNE. The SVM decision boundary was identified by Gaussian Kernel function. **D** The return chart shows the numbers of altered transition types in the CRS-M and CSDS-M groups when compared to the Control-M group. Transition types with a significant increase (CRS-*increase*) or decrease (CRS-*decrease*) in the CRS-M group, with a significant increase (CSDS-*increase)* or decrease (CSDS-*decrease*) in the CSDS-M group, with a significant increase (CRS&CSDS-*increase*) or decrease (CRS&CSDS-*decrease*) in both CRS-M and CSDS-M groups, and with no change (No change) compared with Control-M. **E** The number of total behavioral transitions observed in the Control-M, CRS-M and CSDS-M groups. Statistics in D-E: Kruskal-Wallis test followed by Dunn's post hoc multiple comparisons test, means ± SEM, *$p < 0.05$, **$p < 0.01$, ***$p < 0.001$, ****$p < 0.0001$

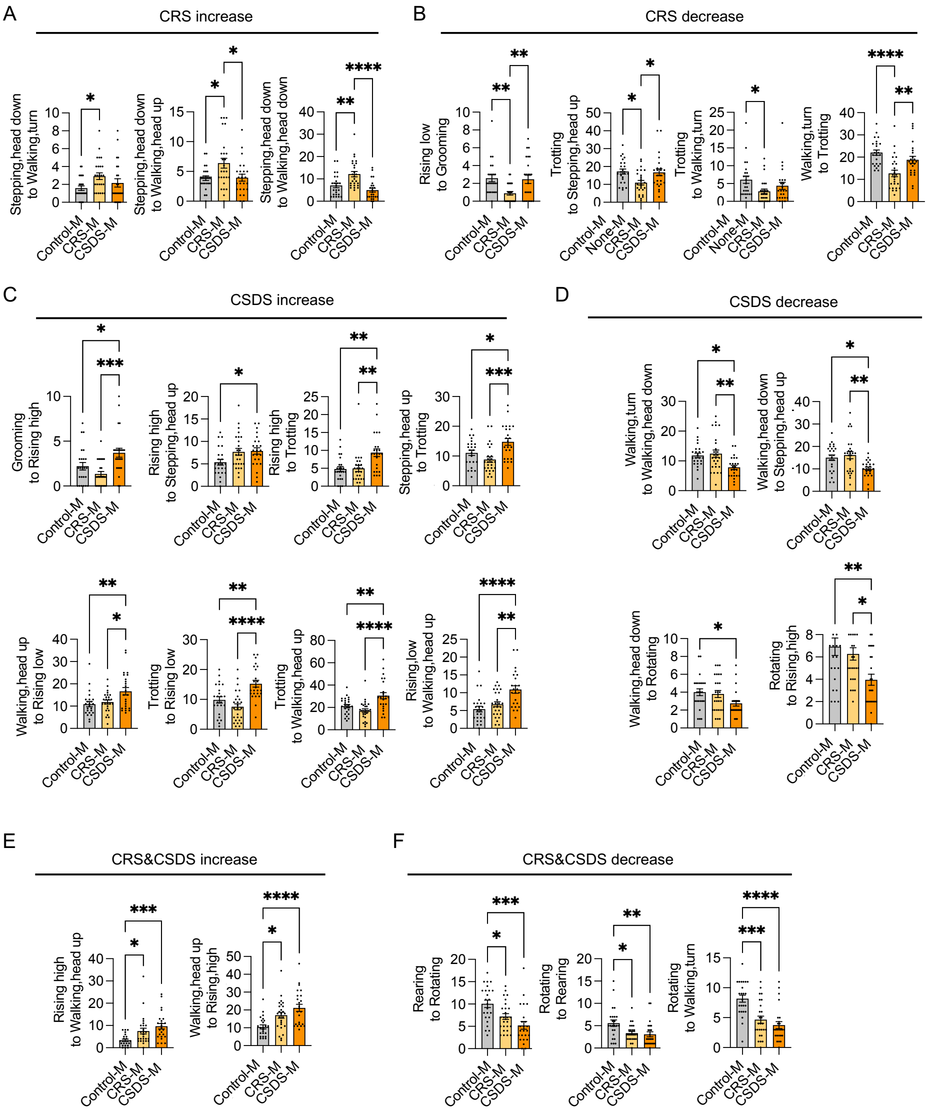

Supplementary Figure 4. Representative behavioral transitions after chronic stress

Comparisons of the numbers of different behavioral transitions in control-M、CRS-M and CSDS-M groups. Transition types with a significant increase (A) or decrease (B) in CRS-M, with a significant increase (C) or decrease (D) in CSDS-M, with a significant increase (E) or decrease (F) in both CRS-M and CSDS-M compared with None-M. Kruskal-Wallis test followed by Dunn's post hoc multiple comparisons test, *$p < 0.05$, **$p < 0.01$, ***$p < 0.001$ ****$p < 0.0001$.

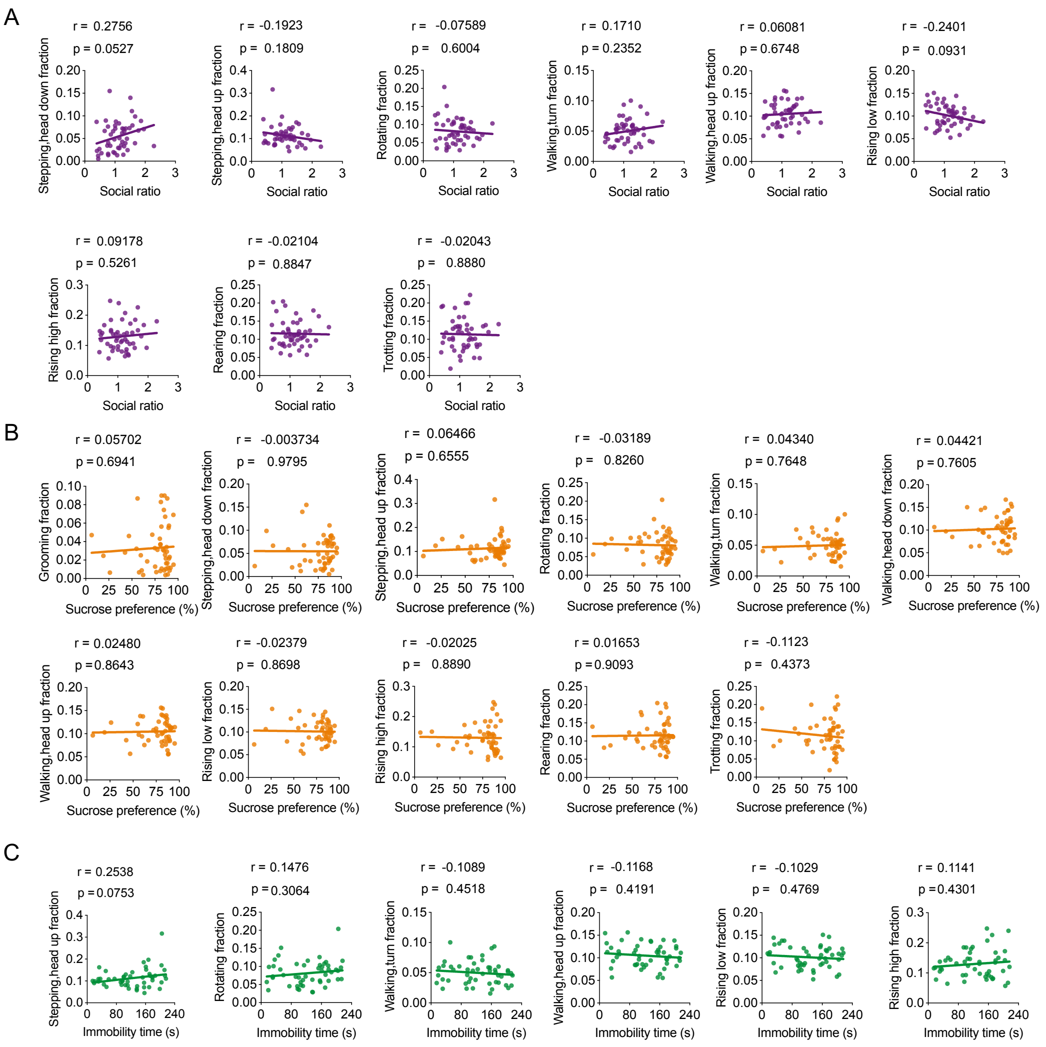

Supplementary Figure 5. Correlation analysis of the fractions of spontaneous behavioral movements with depressive-like behaviors

A-C, Pearson correlation analysis of the fraction of each behavior movement with Social ratio (A), Sucrose preference (B), or Immobility time (C). R indicates the correlation coefficient. CRS n = 26 mice, CSDS n = 24 mice, total 50 mice. Correlation analysis.

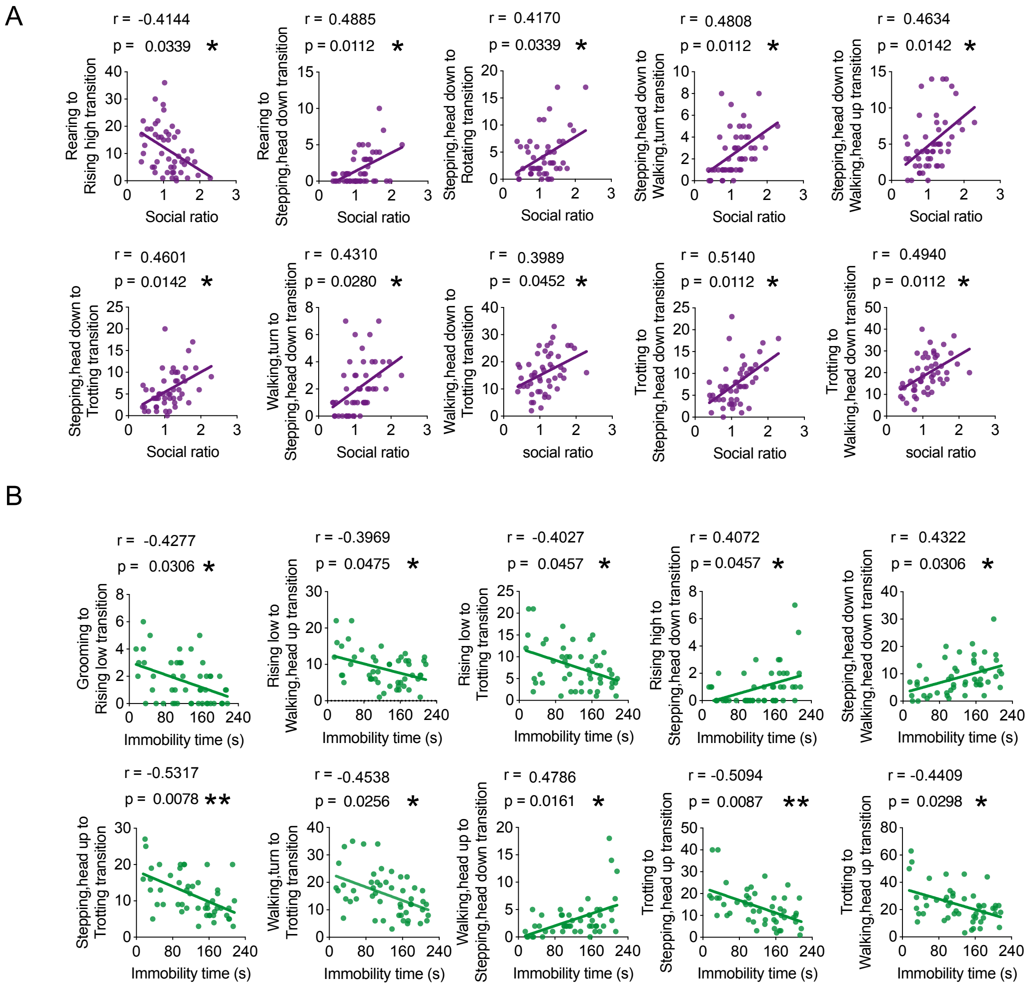

Supplementary Figure 6. Correlation analysis of the machine learning-observed behavioral transitions with depression-like behaviors

A-B, Pearson correlation analysis of the numbers of distinct transition type with Social ratio (A) or Immobility time (B). R indicates the correlation coefficient. CRS n = 26 mice, CSDS n = 24 mice, total 50 mice. Correlation analysis followed by false discovery rate (FDR) corrected. *$p < 0.05$, **$p < 0.01$, ***$p < 0.001$, ****$p < 0.0001$.

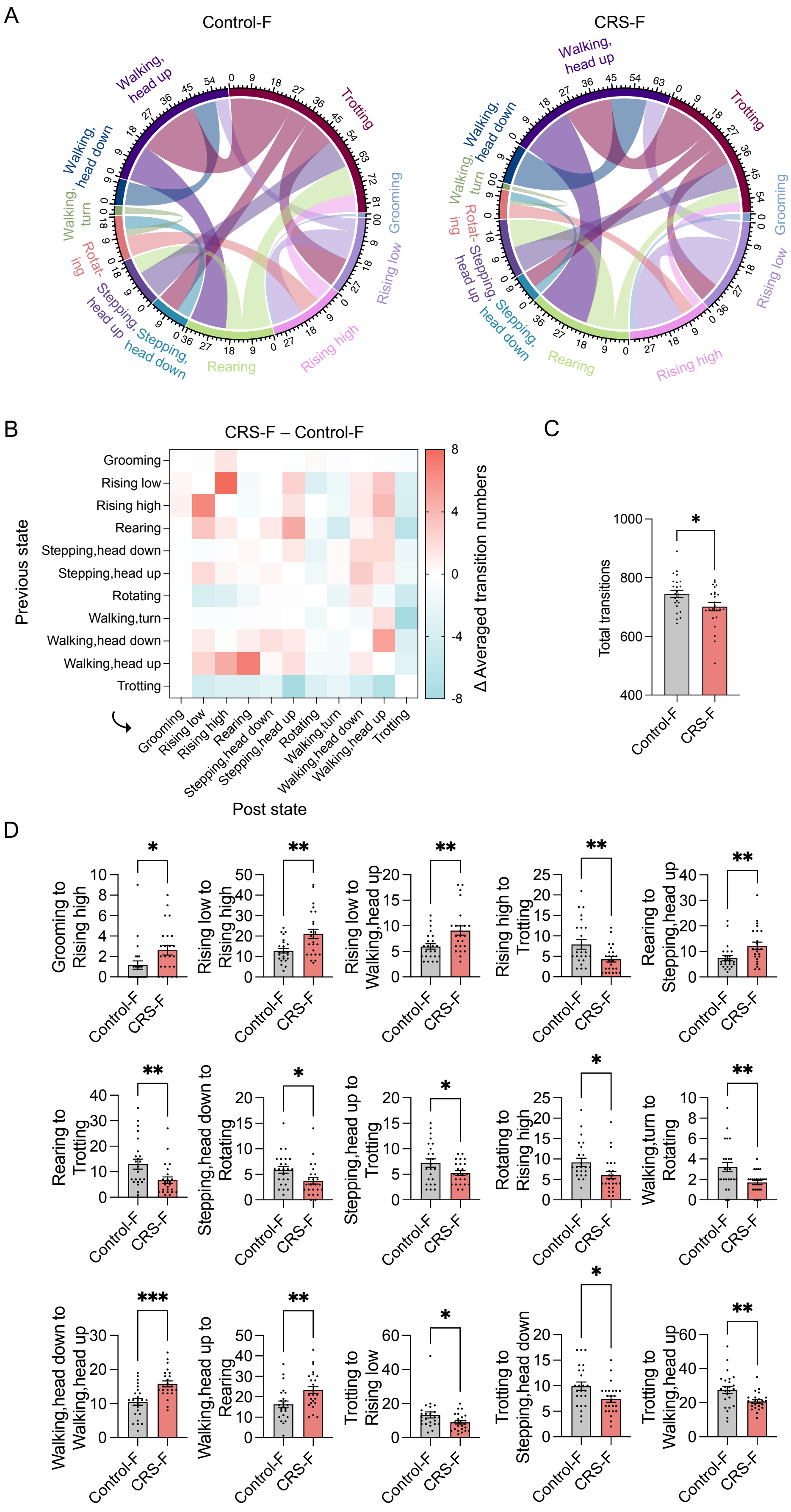
A
Control-F
CRS-F
Trotting
Grooming
Rising low
Rising high
Rearing
Stepping, head down
Stepping, head up
Rotating
Walking, turn
Walking, head down
Walking, head up
B
CRS-F – Control-F
Previous state
Post state
Δ Averaged transition numbers
C
Total transitions
Control-F
CRS-F
D
Grooming to Rising high
Rising low to Rising high
Rising low to Walking,head up
Rising high to Trotting
Rearing to Stepping,head up
Rearing to Trotting
Stepping,head down to Rotating
Stepping,head up to Trotting
Rotating to Rising high
Walking,turn to Rotating
Walking,head down to Walking,head up
Walking,head up to Rearing
Trotting to Rising low
Trotting to Stepping,head down
Trotting to Walking,head up

Supplementary Figure 7. Spontaneous behavioral changes elicited by predator stress in female mice.

A Representative behavioral transitions of Control-F and CRS-F. **B** Comparison of the numbers of behavioral transitions between CRS-F and Control-F groups. The previous behavioral states are listed on the Y-axis and the post states on the x-axis. Δ Averaged transition numbers were calculated using the averaged numbers of each transition type in the CRS-F group subtracted from the Control-F group. Red and blue colors indicate increased and decreased transition numbers, respectively. A total of 106 types of transitions were observed. **C** The number of total transitions in the Control-F and CRS-F groups. **D** Comparisons of the numbers of different behavioral transitions in Control-F and CRS-F groups. Means ± SEM, two-sided Mann–Whitney test. $*p < 0.05$, $**p < 0.01$, $***p < 0.001$, $****p < 0.0001$.

Supplementary Table 1. Statistics

Fig	Comparison	Shapiro–Wilk normality test (alpha = 0.05)	F-test	Analysis	P-value	N
1B (left)	Sucrose consumption (%) in the SPT test	Failed	F (4, 149) = 8.654, P<0.0001	Kruskal-Wallis test followed by Dunn's post hoc multiple comparisons test	Control-M vs. CRS-M: P=0.0013; Control-M vs. CSDS-M: P=0.0020; Control-F vs. CRS-F: P=0.0007	Control-M n=34; CRS-M n=30; CSDS-M n=30; Control-F n=30; CRS-F n=30
1B (right)	Total consumption in the SPT test	Passed	F (4, 149) = 1.294, P=0.2750	One-way ANOVA followed by Tukey post hoc multiple comparisons test,	P>0.05 for each comparison	Control-M n=34; CRS-M n=30; CSDS-M n=30; Control-F n=30; CRS-F n=30
1C (left)	Social interaction ratio in the SI test	Failed	F (4, 149) = 7.894, P<0.0001	Kruskal-Wallis test followed by Dunn's post hoc multiple comparisons test	Control-M vs. CRS-M: P=0.0117; Control-M vs. CSDS-M: P<0.0001; Control-F vs. CRS-F: P=0.0175	Control-M n=34; CRS-M n=30; CSDS-M n=30; Control-F n=30; CRS-F n=30
1C (right)	Durations in the SI zone without/with target mouse present	Passed	Interaction: F (4, 298) = 4.544, P=0.0014; With/Without target: F (1, 298) = 0.4673, P=0.4947; Stress: F (4, 298) = 4.663, P=0.0012	Kruskal-Wallis test followed by Dunn's post hoc multiple comparisons test	No target:Control-M vs. Target:Control-M: P=0.0277; Target:Control-M vs. Target:CRS-M: P=0.0369; Target:Control-M vs. Target:CSDS-M: P=0.0002; Target:Control-F vs. Target:CRS-F: P=0.0177	Control-M n=34; CRS-M n=30; CSDS-M n=30; Control-F n=30; CRS-F n=30
1D	Immobility time in the TST test	Passed	F (4, 149) = 11.34,P<0.0001	Kruskal-Wallis test followed by Dunn's post hoc multiple comparisons test	Control-M vs. CRS-M: P<0.0001; Control-M vs. CSDS-M: P=0.0296 ; CRS-M vs. CSDS-M: P=0.0287; Control-F vs. CRS-F: P-0.0349	Control-M n=34; CRS-M n=30; CSDS-M n=30; Control-F n=30; CRS-F n=30
2B	Fractions of different movement types of Control-M, CRS-M and CSDS-M mice	Passed	Interaction: F (20, 781) = 7.759, P<0.0001; Fractions: F (10, 781) = 73.43, P<0.0001; Stress: F (2, 781) = 0.004010, P=0.9960	Kruskal-Wallis test followed by Dunn's post hoc multiple comparisons test	Marked with asterisk in figure	Control-M n=24; CRS-M n=26; CSDS-M n=24
4A	Fractions of spontaneous behavioral types of Control-F and CRS-F mice	Passed	Interaction: F (10, 506) = 6.611, P<0.0001; Fractions: F (10, 506) = 60.58, P<0.0001; Stress: F (1, 506) = 1.186e-010, P>0.9999	Kruskal-Wallis test followed by Dunn's post hoc multiple comparisons test	Marked with asterisk in figure	Control-F n=24; CRS-F n=24
5B	Fractions of each spontaneous behavioral type in Control+Vehicle, CRS+Vehicle and CRS+Fluoxetine mice	Passed	Interaction: F (20, 561) = 5.285, P<0.0001; Fractions: F (10, 561) = 47.23, P<0.0001; Stress: F (2, 561) = 0.1234, P =P=0.8839	Kruskal-Wallis test followed by Dunn's post hoc multiple comparisons test	Marked with asterisk in figure	Control+Vehicle (male: n=10; female: n=8); CRS+Vehicle (male: n=8; female: n=10); CRS+Fluoxetine (male: n=10; female: n=8)
5D	Representative behavioral transitions observed in the Control+vehicle, CRS+vehicle and CRS+Fluoxetine groups	From left to right: failed, failed, passed, failed	From left to right: F (2, 51) = 10.40, P=0.0002; F (2, 51) = 11.48, P<0.0001; F (2, 51) = 6.668, P=0.0027; F (2, 51) = 9.767, P=0.0003	Kruskal-Wallis test followed by Dunn's post hoc multiple comparisons test	Marked with asterisk in figure	Control+Vehicle (male: n=10; female: n=8); CRS+Vehicle (male: n=8; female: n=10); CRS+Fluoxetine (male: n=10; female: n=8)
